# Beyond Atherosclerosis: Takayasu Arteritis Presenting as Ischemic Stroke in a Young Patient With Significant Traditional Vascular Risk Factors

**DOI:** 10.7759/cureus.85266

**Published:** 2025-06-03

**Authors:** Roohi Khan, Saugata Das, Nasar Ahmad

**Affiliations:** 1 Stroke Medicine, The Royal Wolverhampton NHS Trust, Wolverhampton, GBR

**Keywords:** cerebrovascular disease, corticosteroids, immunosuppression, stroke, ta, takayasu arteritis, takayasu vasculitis, vasculitis

## Abstract

A 46-year-old lady, with insulin-dependent type 2 diabetes, ischaemic heart disease, hypertension, high cholesterol, and being a current smoker, presented with acute left-sided weakness involving her upper and lower limbs. She was admitted with a clinical diagnosis of right lacunar ischemic stroke. Initial CT brain revealed an old right basal ganglia infarct, and CT carotid angiography revealed irregularities in the right middle cerebral artery with no occlusion, subtle mural thickening, and periarterial changes surrounding the origin of the great vessels arising from the aortic arch, features suggestive of vasculitis. Subsequent MRI brain demonstrated multiple infarcts in the high right frontoparietal and occipital regions, while CT aorta with contrast revealed mural thickening in the aortic arch involving the proximal aspect of the great vessels and stenosis of the proximal left subclavian artery, including the vertebral artery origin. Evidence of atheroma in the right coronary artery and iliac vessels was also noted. Dual antiplatelet therapy (DAPT) was initiated, but given the characteristic vascular imaging features of a thickened aortic wall and its branches, the diagnosis of Takayasu arteritis (TA) was made following rheumatology consultation. Unfortunately, she did experience a further ischaemic stroke on the same admission, but recovered well following immunosuppression. Treatment with steroids was initiated, and following a subsequent review in a specialist UK vasculitis centre, she was commenced on methotrexate, following which she has not had any further recurrent events. This case highlights the challenges of diagnosing TA in stroke patients with multiple comorbidities with traditional vascular risk factors and the need for a low threshold of suspicion in those with atypical imaging, particularly angiographic findings, especially if they are relatively young. A timely diagnosis of arteritis could help facilitate targeted management to reduce future risks and recurrent vascular episodes.

## Introduction

Takayasu arteritis (TA), also known as “pulseless disease,” is a rare and chronic granulomatous inflammatory disease that affects predominantly the large arteries, primarily the aorta and its major branches. It is more common in the Asian population, though reported incidence in Olmsted County in the US has been quoted as 2.6 per million. It is, however, considered that the actual prevalence is often underestimated based on autopsy studies in Japan. It is recognized as an autoimmune disorder, leading to inflammation, resulting in serious complications, such as arterial narrowing or blockages, organ damage, and increased risk of cardiovascular and cerebrovascular ischemic events such as stroke [1].

Our case report highlights a young patient with typical and traditional vascular risk factors, such as type 2 diabetes, hypertension, ischemic heart disease, dyslipidemia, and being an active smoker experiencing recurrent ischemic strokes due to underlying large vessel vasculitis subsequently diagnosed as TA based on characteristic vessel wall thickening on imaging and multi-specialty input. This case aims to raise awareness of imaging characteristics of TA, which is different from traditional atherosclerotic changes, and recognition of this should alert clinicians to any potential alternative etiology when assessing acute stroke patients at the front door.

## Case presentation

A 46-year-old female presented to the emergency department with left upper and lower limb weakness, beyond the thrombolysis window, as a wake-up stroke. Her initial National Institutes of Health Stroke Scale (NIHSS) score was 9 with dense left hemiparesis and decreased sensation on the left side. Notable past medical history included poorly controlled type 2 diabetes (HbA1c: 110 mmol/mol, approximately 12.2%) on high-dose insulin and oral hypoglycemic agents, hypertension, dyslipidemia, significant active smoking history of 20 pack years, and a history of non-ST elevation myocardial infarction (MI) in 2018. Of note, she also had a past history of Guillain-Barré syndrome in 2015 with subsequent good recovery, which did not have any bearing on her current presentation.

**Figure 1:**
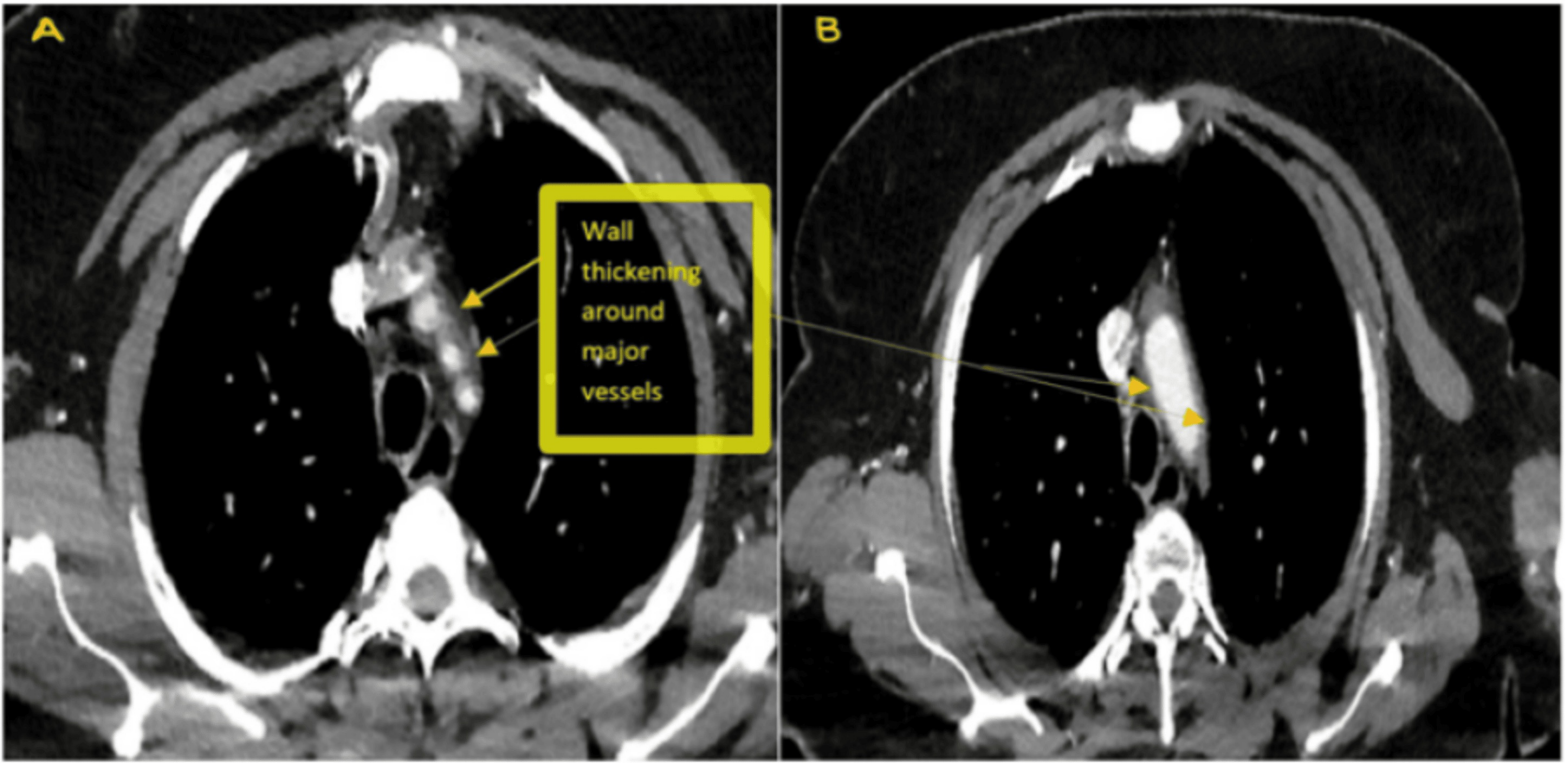
Initial CT angiography showing wall thickening around the major vessels

**Figure 2:**
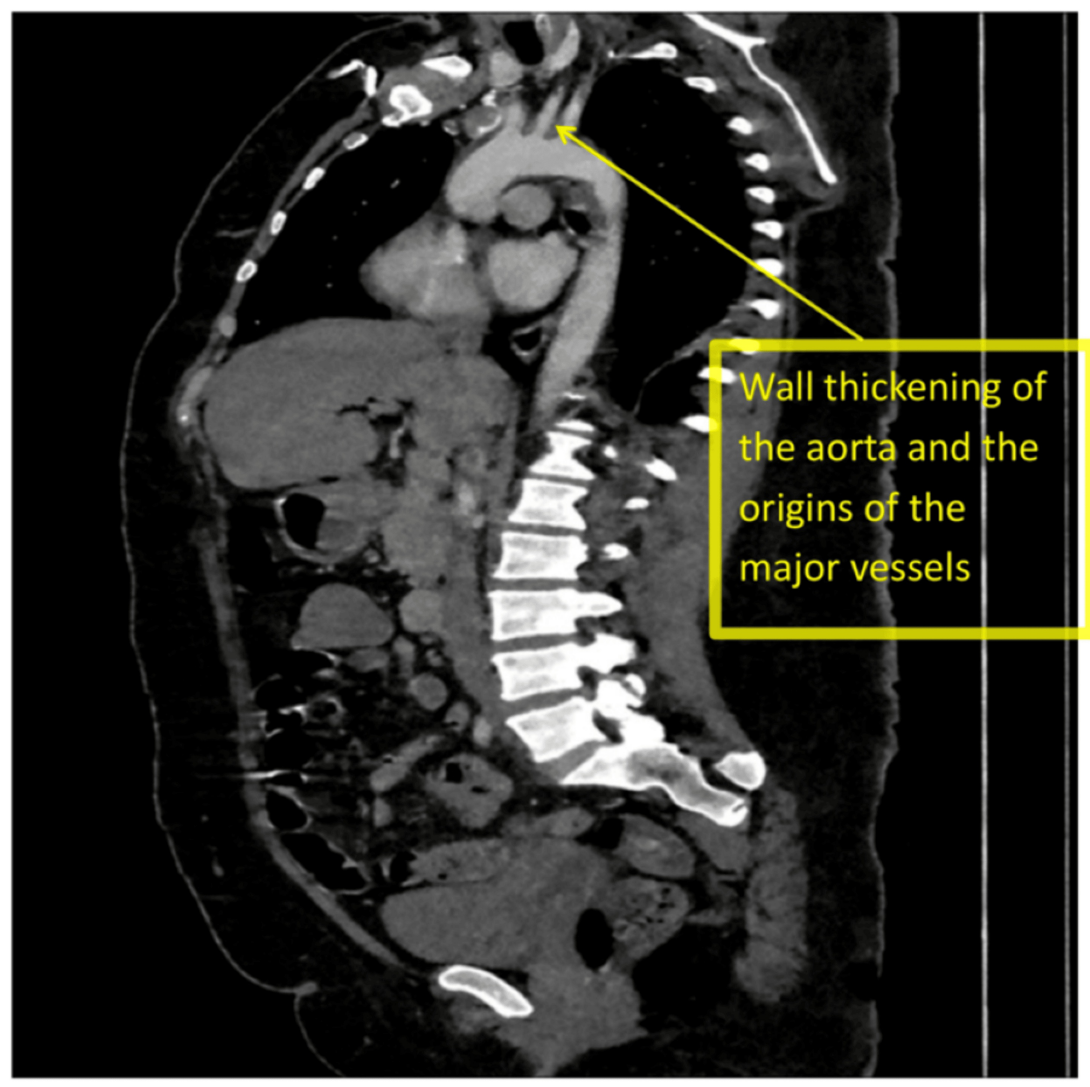
Sagittal section of CT aortography showing wall thickening of the aorta and the origins of the left subclavian, common carotid, and right brachiocephalic artery

**Figure 3:**
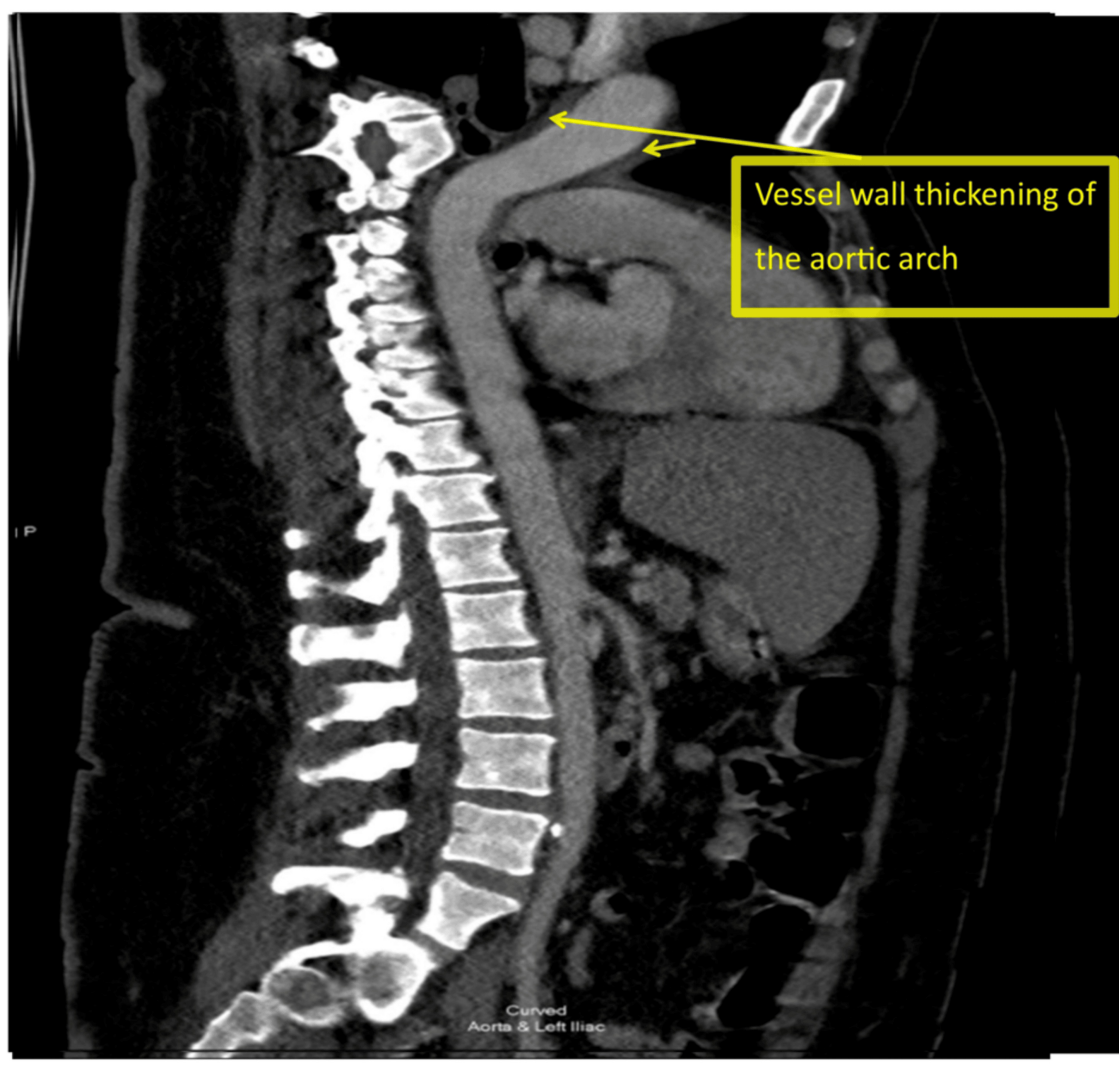
Sagittal section of CT aortography showing vessel wall thickening of the aortic arch

**Figure 4:**
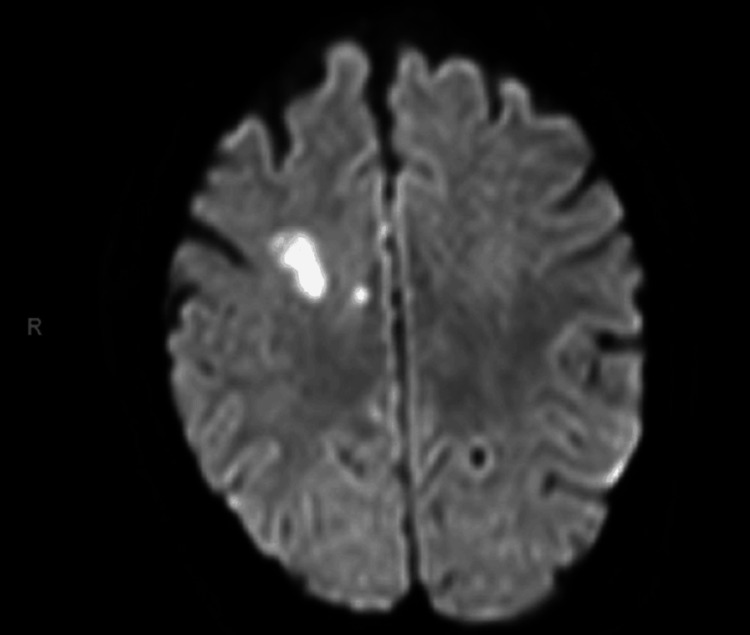
Initial MRI brain showing acute infarcts in the right high frontoparietal lobe and few other tiny infarcts of varying ages with restriction diffusion on DWI/ADC scans DWI/ADC: Diffusion-weighted imaging/apparent diffusion coefficient

Initial CT brain showed old right basal ganglia infarcts. CT carotid angiography revealed irregularities in the right middle cerebral artery with no occlusion, subtle mural thickening, and periarterial changes surrounding the origin of the great vessels arising from the aortic arch, suggestive of stenosis (Figure 1A and Figure 1B). CT aorta with contrast showed features consistent with TA (mural thickening of great vessels and stenosis of proximal left subclavian artery) (Figure 2 and Figure 3). MRI brain confirmed multiple acute infarcts in the high right frontoparietal and occipital regions (Figure 4).

**Figure 5:**
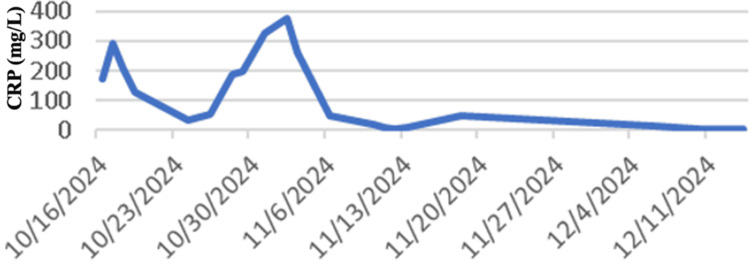
Graph A - CRP levels throughout admission X-axis: Inflammatory markers (CRP); Y-axis: Dates of tests

**Figure 6:**
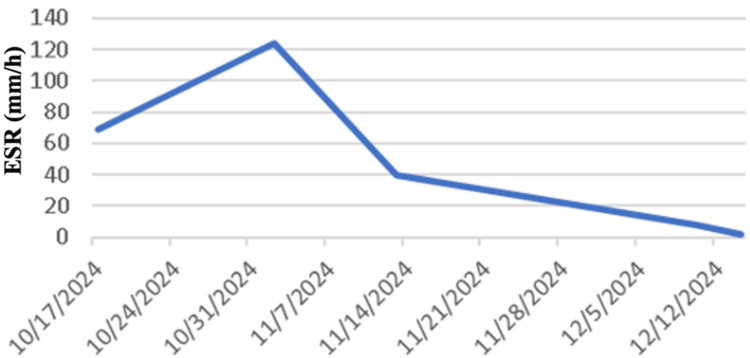
Graph B - ESR levels throughout admission X-axis: Inflammatory markers (ESR); Y-axis: Dates of tests

The patient was admitted with an initial diagnosis of right lacunar ischemic stroke and started on dual antiplatelets. ECG at the time of admission showed non-specific ST changes with mildly elevated troponin with subsequent normalization. She had no symptoms of chest pain, palpitations, or shortness of breath. An echocardiogram suggested normal left ventricular function with no regional wall motion abnormality but evidence of concentric left ventricular hypertrophy with mild diastolic dysfunction, consistent with long-standing hypertension. It was also noted that her inflammatory markers were elevated at the time of admission. However, during admission, she was also found to have a dental abscess that required incision and drainage and initiation of antibiotics. This improved her inflammatory markers initially, but there was a sharp rise noted again in a few days (Figure 5 and Figure 6).

**Figure 7:**
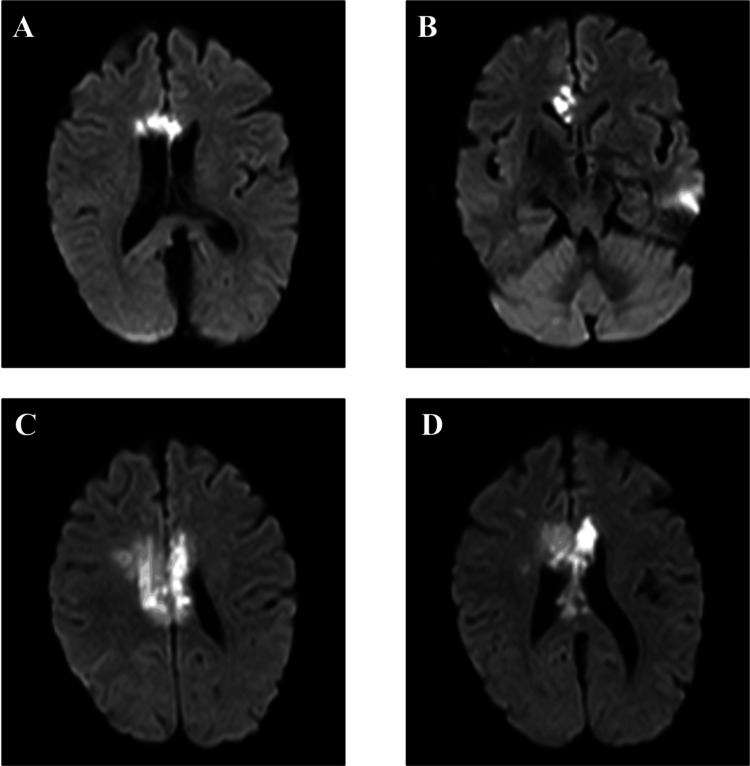
Repeat MRI showing new areas of callosal and pericallosal with high signal change bilaterally with restriction diffusion on DWI/ADC scans DWI/ADC: Diffusion-weighted imaging/apparent diffusion coefficient

In view of the characteristic vessel imaging and presentation with multiple infarcts, with rising inflammatory markers, with no other plausible etiology, a diagnosis of large vessel vasculitis was made following consultation with the rheumatologist. A PET scan was considered but delayed due to logistics and other issues. Therefore, prednisolone 40 mg once daily was started in view of high clinical suspicion. When her steroids were stopped for a brief period during the time of her scheduled PET CT scan, unfortunately, she had a further vascular event with new infarcts confirmed on MRI brain imaging (Figures 7A-7D), necessitating recommencement of high-dose steroids. Vessel wall MRA imaging was not available in our local centre.

The patient had reported no underlying constitutional symptoms, including low-grade pyrexia or history of claudication, or any family history of significance. There was an audible bruit noted in the left subclavian artery that corresponds to the stenosis noted on imaging, while no difference was noted in her BP in either arm (right arm: 144/86 mm of Hg; left arm: 150/ 88 mm of Hg). However, atypically, she was not noted to have any absent or reduced peripheral pulses.

Relevant investigations during the admission showed negative anti-neutrophil cytoplasm antibodies (ANCA), weakly positive antinuclear antibody (ANA), not deemed to be of clinical significance, and negative extractable nuclear antigens (ENA) and anti-double-stranded DNA (dsDNA). Hepatitis B and C and HIV screens were negative, as were cardiolipin, beta 2 glycoprotein, and lupus anticoagulant, thus excluding antiphospholipid syndrome. She had negative blood cultures; screening tests for tuberculosis and syphilis were negative. An ambulatory 24-hour ECG done on admission suggested sinus rhythm throughout.

Following treatment with steroids, a further MRA aorta with contrast did not show any appreciable previously seen thickening around the large vessels, but the left subclavian stenosis seen on the previous CT scan was still noted. A delayed PET CT did not detect any activity, given that she was on high-dose steroids for five weeks, which also led to normalizing of her previously elevated inflammatory markers. These results, along with near complete resolution of the initial CT aorta findings, added to the complexity of this case, though can also be explained as masking by immunosuppression.

The patient's clinical presentation and initial scans suggested vessel wall thickening of the aorta and origin of left subclavian, which are not associated with typical atherosclerotic disease, were considered highly suspicious for large vessel vasculitis, specifically Takayasu’s arteritis. As per the 2022 classification criteria established by the American College of Rheumatology and the European Alliance of Associations for Rheumatology (EULAR), which are used to identify TA, the scoring for our patient is 8. Improved radiographic appearances and inflammatory markers following treatment with steroids lent support to the initial diagnosis.

Her case was discussed at the specialist vasculitis centre in Hammersmith Hospital, London (Imperial College Healthcare NHS Trust), where the probable diagnosis was confirmed, and it was recommended that she be started on methotrexate to control the inflammatory process with gradual tapering of prednisolone. A further plan to consider mycophenolate mofetil and tocilizumab in the future, depending on her progress, with follow-up appointments with the rheumatologist, was in place. Clinically, at the time of discharge, she was able to eat and drink normally with residual weakness on her left side, with mild dysarthria and requiring a hoist to sit in a supportive chair. She declined further in-patient rehabilitation and preferred to go home with a package of care.

## Discussion

The medical condition known as TA received its name from Japanese ophthalmologist Mikito Takayasu during his initial description in 1908. It typically affects young women, especially those of Asian descent. Stenosis, occlusions, and aneurysmal dilatations are observed in late stages of the disease [2]. The conditions of stenosis and occlusion are more frequent in patients from Europe, the United States, and Japan, while aneurysms mainly affect patients in India, Thailand, Mexico, and African nations [3].

Chief symptoms of TA begin with general symptoms that include fever, malaise, weight loss, and anorexia before specific manifestations occur. The symptoms of arterial insufficiency may also manifest as hypertension, together with neurologic problems and upper limb pain due to claudication.

The definite etiology of the disease is still unestablished. The main characteristic of this condition is inflammation, and some experts believe that cell-mediated immunity causes the disease. The eventual development of transmural fibrous thickening in arterial walls results in ischemic changes that form a pseudoaneurysm [4]. The inflammatory process affects the vasa vasorum and the medio-adventitial junction. Infiltration by mononuclear cells, along with potential giant cell granulomatous reaction, is often a key feature [5].

Environmental and genetic factors are also thought to play roles in the development of the disease. Genetic screening has shown polymorphisms in IL-12, IL-6, and IL-2 genes in a population of Turkish patients with TA. HLA-Bw5 and HLA-B39.2 are reportedly increased in frequency in some populations [1].

Diagnosis

The 2022 classification criteria established by the American College of Rheumatology and EULAR are used to identify TA in patients already diagnosed with medium or large vessel vasculitis. These criteria require that alternative mimicking conditions be excluded before application. Classification begins with two mandatory conditions: the individual must be 60 years of age or younger at the time of diagnosis and must show imaging-confirmed evidence of vasculitis. Additional clinical indicators contribute to a weighted score, including female sex, ischemic cardiac symptoms such as angina, limb claudication, audible vascular bruits, diminished upper limb pulses, abnormalities of the carotid artery, and a systolic blood pressure difference greater than 20 mmHg between arms. Imaging features further increase the score based on the number of affected arterial territories, bilateral arterial involvement, and the presence of aortic damage extending to renal or mesenteric vessels. A combined score of 5 or more from these parameters is necessary to confirm the classification of TA under these guidelines [6].

Vascular imaging also plays an important role in the diagnosis. Computed tomography angiography (CTA) and magnetic resonance arteriography (MRA) represent non-invasive imaging methods that healthcare providers often use, in addition to fluorodeoxyglucose positron emission tomography (FDG-PET), which helps evaluate vascular inflammation and determines disease activity using integrated indices [7,8]. Better disease pathogenesis becomes possible through morpho-pathology investigations because inflammation affects all artery layers, resulting in elastic tissue damage along with new blood vessel development in the intima and media [8]. Triggers can be infections such as tuberculosis, syphilis, varicella zoster, measles, or other underlying autoimmune conditions [9].

A healthcare provider must suspect TA of an accurate diagnosis, thus requiring detailed clinical examination and interview. CTA serves as the diagnostic standard for primary distribution staging of TA according to the 1994 Tokyo International Conference Classification due to its capability to show vessel wall thickening and luminal narrowing [5].

Angiographic Classification of TA

The classifications depend on which blood vessels become involved during angiographic assessments, as summarized in Table 1 [10].

**Table 1:** Angiographic classification of Takayasu’s arteritis

Type	Typical blood vessel involvement
I	Affecting branches of aortic arch
IIa	Involving the ascending aorta, aortic arch and its branches
IIb	Involving the ascending aorta, aortic arch and its branches and thoracic descending aorta
III	Affecting the thoracic descending aorta, abdominal aorta and/or renal arteries
IV	Affecting the abdominal aorta and/or renal arteries
V	Presenting with features from Types IIb and IV

Role of FDG-PET in Diagnosis

The diagnostic value and disease activity assessment in large vessel vasculitis can be enhanced by using combined functional FDG-PET scans with anatomical CT angiography, known as FDG PET/CT(A) [11]. In elderly patients, the atherosclerotic vascular uptake reduces the ability of 18F-FDG PET to specifically diagnose vasculitis. CT imaging with calcifications, along with 18F-FDG uptake patterns, serves to differentiate between the two conditions. The pattern of uptake for large vessel vasculitis consists of diffuse linear patterns, while atherosclerosis exhibits typical patchy patterns [12].

18F-FDG PET interpretation criteria use both visual and semiquantitative parameters, but there is insufficient evidence that semiquantitative parameters outperform a visual grading scale. A standardized 4-point visual grading scale (arterial to liver uptake) is used, with grade 0, no uptake; grade 1, lower than liver uptake; grade 2, similar to liver uptake; and grade 3, higher than liver uptake. The visual grading scale allows evaluation of individual arterial segments that result in a quantified PET vascular activity score (PETVAS) between several segments (typically 7-15). The overall assessment of disease burden can offer a better evaluation of treatment response because it provides a comprehensive evaluation of disease burden. 18F-FDG uptake in cranial arteries is scored as a 3-point visual grading (0-2), with grade 0 representing uptake not above the surrounding tissue, grade 1 representing uptake slightly above the surrounding tissue, and grade 2 representing uptake significantly above the surrounding tissue [13].

Though hyperglycemia does not significantly impact detecting inflammatory lesions in 18F-FDG PET, a negative correlation has been observed between blood glucose levels and 18F-FDG arterial wall uptake. Lower blood glucose levels around 7 mmol/L are preferred for better interpretation, and hence oral steroid therapy, even for 10 days, decreases the sensitivity of 18F-FDG PET for diagnosing large vessel vasculitis. The test sensitivity remains unaffected when it is conducted within three days of initiating oral glucocorticoid treatment. The increase of liver uptake caused by steroids creates challenges in visual assessment of vascular 18F-FDG uptake [14].

The management of TA receives guideline recommendations from both the EULAR and the American College of Rheumatology [15,16]. The treatment of TA heavily depends on systemic steroid medications. Mostly, diseases such as TA are treated with disease-modifying anti‐rheumatic drugs combined with azathioprine and methotrexate to decrease steroid dependency while minimizing steroid-related adverse effects. Tocilizumab and anti-tumour necrosis factor inhibitors, along with tocilizumab, represent new biologic agents that doctors consider for early treatment of TA while seeking to prevent disease relapses. Janus kinase (JAK) inhibitors such as tofacitinib and upadacitinib, along with T cell activation inhibitor (abatacept), have been studied but do not form the core of standard treatment [1].

Transluminal angioplasty provides limited help in treating patients who have TA. The fibrous texture of arterial obliteration blocks the possibility of lasting long-term improvements [17]. Traditionally, professionals reserved surgery for patients who experienced active symptoms of arterial occlusive disease that did not respond to corticosteroids and immunosuppressive treatments and now consider surgery for aneurysm management and aortic regurgitation treatment, as well as arterial stenosis or dilation [18]. The alternative surgical intervention for TA patients is known as arterial reconstruction. The reconstruction procedure depends on lesion locations combined with each patient's surgical anatomy characteristics [19].

The disease progression remains uncertain because relapses occur frequently, and real-time assessment of long-term TA outcomes has not been thoroughly researched. The rarity of TA and its heterogeneous presentation, progression, and response to treatment typically results in delayed diagnosis and extensive vessel injury due to inflammation and stenosis. Most patients require repeated and prolonged courses of therapy due to relapses. Although mortality is not high, substantial morbidity occurs in most patients due to delayed diagnosis and resistant disease. Clinical presentation varies from asymptomatic to serious neurovascular manifestations, and stroke is reported as the first symptom in 6-8% of TA and can occur during the course of the disease in around 16-20% of the patients [20]. This can be challenging, especially in those with other risk factors for atherosclerosis and stroke.

Our case depicts a patient with a life-changing stroke as a complication of TA, in addition to several other traditional atherosclerotic vascular risks. In addition to fulfilling both criteria of absolute requirements for diagnosis due to age and imaging features, the score based on the recent American College of Rheumatology and EULAR classification for our case was 8 (gender: 1, history of ischemic cardiac pain: 2, vascular bruit: 2, arterial territories: 3). A multidisciplinary approach involving rheumatologists, stroke physicians, and neuroradiologists was key to early diagnosis, management, and follow-up. A collaborative approach between healthcare professionals can ensure prompt diagnosis and enhance both the quality of life and outcome for such patients.

## Conclusions

TA is a relatively rare inflammatory large vessel vasculitis. Though ischaemic stroke is quite uncommon as the first presentation of TA, clinical suspicion based on presentation and vessel imaging, as in our case, can lead to early diagnosis. This provides an opportunity for timely interventions to minimize inflammation and its progression to prevent permanent stenotic changes to large vessels and end-organ damage. The coexistence of TA and atherosclerosis in the same patient with vascular risk factors may pose a significant diagnostic and therapeutic challenge. Our case report emphasizes the importance of considering large vessel vasculitis, such as TA, in relatively younger patients presenting with an acute vascular episode and atypical angiographic features despite other co-existing vascular risks. The role of advanced imaging and involvement of a multidisciplinary team at the earliest opportunity to strategize management and facilitate a favourable outcome in such patients is always crucial.
